# Dendritic Cell-Specific Deletion of β-Catenin Results in Fewer Regulatory T-Cells without Exacerbating Autoimmune Collagen-Induced Arthritis

**DOI:** 10.1371/journal.pone.0142972

**Published:** 2015-11-20

**Authors:** C. Henrique Alves, Julia L. Ober-Blöbaum, Inge Brouwers-Haspels, Patrick S. Asmawidjaja, Adriana M. C. Mus, Wida Razawy, Marlieke Molendijk, Björn E. Clausen, Erik Lubberts

**Affiliations:** 1 Department of Rheumatology, Erasmus MC, University Medical Center, Rotterdam, The Netherlands; 2 Department of Immunology, Erasmus MC, University Medical Center, Rotterdam, The Netherlands; 3 Institute for Molecular Medicine, University Medical Center of the Johannes Gutenberg-University Mainz, Mainz, Germany; University Hospital Jena, GERMANY

## Abstract

Dendritic cells (DCs) are professional antigen presenting cells that have the dual ability to stimulate immunity and maintain tolerance. However, the signalling pathways mediating tolerogenic DC function *in vivo* remain largely unknown. The β-catenin pathway has been suggested to promote a regulatory DC phenotype. The aim of this study was to unravel the role of β-catenin signalling to control DC function in the autoimmune collagen-induced arthritis model (CIA). Deletion of *β-catenin* specifically in DCs was achieved by crossing conditional knockout mice with a *CD11c-*Cre transgenic mouse line. Bone marrow-derived DCs (BMDCs) were generated and used to study the maturation profile of these cells in response to a TLR2 or TLR4 ligand stimulation. CIA was induced by intra-dermal immunization with 100 μg chicken type II collagen in complete Freund’s adjuvant on days 0 and 21. CIA incidence and severity was monitored macroscopically and by histology. The T cell profile as well as their cytokine production were analysed by flow cytometry. Lack of *β-catenin* specifically in DCs did not affect the spontaneous, TLR2- or TLR4-induced maturation and activation of BMDCs or their cytokine production. Moreover, no effect on the incidence and severity of CIA was observed in mice lacking *β-catenin* in CD11c^+^ cells. A decreased frequency of splenic CD3^+^CD8^+^ T cells and of regulatory T cells (Tregs) (CD4^+^CD25^high^FoxP3^+^), but no changes in the frequency of splenic Th17 (CCR6^+^CXCR3^-^CCR4^+^), Th2 (CCR6^-^CXCR3^-^CCR4^+^) and Th1 (CCR6^-^CXCR3^+^CCR4^-^) cells were observed in these mice under CIA condition. Furthermore, the expression of IL-17A, IL-17F, IL-22, IL-4 or IFNγ was also not affected. Our data indicate that ablation of *β-catenin* expression in DCs did not alter the course and severity of CIA. We conclude that although deletion of *β-catenin* resulted in a lower frequency of Tregs, this decrease was not sufficient to aggravate the onset and severity of CIA.

## Introduction

Rheumatoid arthritis (RA) is an autoimmune disease characterized by chronic inflammation and destruction of cartilage and bone [1, 2]. Although the etiology of RA has yet to be established, it is believed that RA results from a breach in immune tolerance. Interactions between osteoclasts and immune cells, such as T cells primed by activated dendritic cells (DCs), may contribute to the pathogenesis of RA in humans and murine models [3]. DCs are professional antigen presenting cells that continuously sample their environment for foreign and self-antigens and play a prominent role balancing immunity and tolerance [4, 5]. The role of DCs in the initiation of arthritis was demonstrated in mice, where administration of collagen-pulsed mature DCs is sufficient to induce arthritis. Development of the disease is mediated by both adaptive and innate effects of DCs, namely priming of autoreactive T cells and induction of local inflammation via soluble mediators such as TNF [6]. However, owing to their regulatory function DCs might also have therapeutic potential to treat RA, since administration of semi-mature or tolerogenic DCs can inhibit collagen-induced arthritis (CIA) [7–9]. In this context, it is crucial to dissect the molecular pathways that regulate the balance between pro-inflammatory and tolerogenic functions of DCs. It has previously been suggested that β-catenin, an essential component of the canonical wingless (wnt) pathway and widely expressed in immune cells including DCs, plays an important role in the switch between a tolerogenic and an immunogenic DC phenotype [10, 11]. Canonic β-catenin signalling represents a receptor-mediated signal transduction pathway. Binding of a wnt ligand to its receptor frizzled and the co-receptor lipoprotein receptor-related protein (LRP) 5/6 inhibits the activity of the destruction complex targeting β-catenin for degradation. This leads to the cytoplasmic accumulation of β-catenin and its translocation to the nucleus in order to interact with the T cell-specific transcription factor (TCF) and lymphoid enhancer-binding factor (LEF) that regulate the expression of wnt target genes [10].

Indeed, there is accumulating evidence that the wnt pathway is involved in promoting a tolerogenic DC phenotype. Initially, it was found that disruption of E-cadherin-mediated cell-cell contacts or mechanical agitation of bone marrow-derived DCs (BMDCs) induces a tolerogenic DC phenotype *in vitro*, which is dependent on β-catenin signalling and nuclear translocation [12]. While activation of β-catenin signalling causes ‘phenotypic’ BMDC maturation, these cells fail to produce pro-inflammatory cytokines. Instead, they induce IL-10 production from naïve T cells *in vitro* and protection from experimental autoimmune encephalomyelitis (EAE) in mice [12]. In line, in *CD11c*-specific *β-catenin* knockout mice, lamina propria DCs secrete lower amounts of immunosuppressive cytokines (interleukin-10 (IL-10), and TGF-β) required for regulatory T cell (Treg) production. Rather, inflammatory T helper type-17 (Th17) cells are predominantly generated and, consequently, mice are more susceptible to induction of colitis [13]. Similarly, loss of LRP5/6 β-catenin-mediated signalling in DCs results in increased Th1/Th17 cell differentiation and a reduced Treg response. This is due to increased and decreased production of pro- and anti-inflammatory cytokines, respectively, driving exacerbated autoimmune neuroinflammation (EAE) [14]. Activation of canonical wnt/β-catenin signalling, by TLR2-mediated signals or using a β-catenin agonist, results in reduced frequencies of Th1/Th17 cells, enhanced frequency of Tregs and in the suppression of pro-inflammatory cytokines production limiting chronic autoimmune inflammation [14, 15].

In this study, we subjected mice lacking *β-catenin* specifically in CD11c^+^ DCs to the autoimmune CIA model. In addition to monitoring disease incidence and severity, we also examined the T cell profile during CIA. Our data indicate that specific deletion of *β-catenin* signalling in DCs results in lower Treg differentiation without enhancing the severity of CIA.

## Material and Methods

### Animals and genotyping

All procedures concerning animals were performed with permission of the Committee on the Ethics of Animal Experiments of the Erasmus MC (DEC nr. 128-11-07 and 128-14-08). All mice used were maintained on a C57BL/6 genetic background, kept under specific pathogen-free conditions and provided with food and water *ad libitum* at the Erasmus MC experimental animal facility. Eight to 12 week-old mice were used for all experiments. *β-catenin* conditional knockout mice (*β-catenin* ^*FL/FL*^ *)* [16] were crossed with a *CD11c*-Cre transgenic mouse line [17], to specifically remove *β-catenin* from DCs (*β-catenin* ^*FL/FL*^ */CD11cCre* ^*+/-*^ *)*, mentioned in the text as β-cat^DEL^. Littermates (*β-catenin* ^*FL/FL*^ *)* were used as a control and are mentioned in the text as control.

Genotyping was performed on chromosomal DNA isolated from toe biopsies. The genotyping of the *loxP*-flanked (floxed) *β-catenin* transgenic animals was performed using the following primers: 5’-CACCATGTCCTCTGTCTATCC-3’, 5’-AAGGTAGAGTGATGAAAGTTGTT-3’ and 5’-TACACTATTGAATCACAGGGACTT-3’ resulting in products of 221 bp for the wild-type allele, 324 bp for the floxed allele and 500 bp for the deleted allele. The following primers were used to detect the transgenic *Cre* expression: 5’-CCGGTCGATGCAACGAGTGA-3’ and 5’-GGCCCAAATGTTGCTGGATA-3’ generating a 235 bp product.

### Bone marrow-derived dendritic cells

BMDCs were prepared by flushing the femurs and tibias of β-cat^DEL^ and control mice, filtering through a 100 μm nylon cell strainer (BD Falcon, Sunnyvale, CA, USA), and depleting red blood cells by 1 minute incubation at 4°C with ammonium chloride lysis buffer (NH_4_Cl 0.1555 M, KHCO_3_ 10 mM, Na_2_EDTA.2H_2_O 0.1 mM in H_2_O). Half a million cells/ml were incubated for 10 days in RPMI-1640 with ultraglutamine 1 medium (Lonza, Basel, Switzerland) containing 1% of recombinant mouse granulocyte-macrophage colony stimulating factor (GM-CSF), 5% fetal calf serum (FCS), 100 U/ml Penicillin/Streptomycin and 50 μM β-mercaptoethanol. On day 9 BMDCs were left unstimulated or pulsed for 24 hours with LPS (1 ng/ml; Invitrogen, San Diego, CA, USA), chicken type II collagen (CII) (50 μg/ml; Chondrex, Redmond, WA, USA), LPS (1 ng/ml) and CII (50 μg/ml) or *Mycobacterium tuberculosis* (strain H37Ra; Difco) (25 μg/ml). For CII-specific stimulation, CII was heat inactivated for 10 minutes at 80°C. All the conditions were performed in triplicates. Data are representative of two-three independent experiments (*n* = 6–9 mice/group).

### Cytokine ELISA

The levels of IL-6, IL-10, IL-12p70 and IL-23 cytokines were measured in the supernatants of 10-day cultures of BMDCs that were unstimulated or pulsed for 24 hours in the presence of LPS (1 ng/ml) and CII (50 μg/ml) or *Mycobacterium tuberculosis* (25 μg/ml). Cytokine-specific ELISAs were used and performed according to the manufacturer’s instructions. Undiluted samples were used to detect IL-10, IL-12p70 and IL-23; to detect IL-6 samples were diluted 50 times.

### Collagen-induced arthritis

Mice were immunized with 100 μg (2 mg/ml) chicken type II collagen (Chondrex, Redmond, WA, USA) emulsified in an equal volume of complete Freund’s adjuvant (CFA) containing 1 mg/ml heat killed *Mycobacterium tuberculosis* (strain H37Ra; Difco), administered intradermally (i.d.) in the tail base on day 0 and subcutaneously (s.c.) on the mouse back on day 21. Mice were killed on day 35. Mice were considered to have arthritis when significant changes in redness and/or swelling were noted in the digits or in other parts of the paws. Arthritis was scored visually using the following scale: 0, non-inflamed; 1, mild inflammation; 1.5, marked inflammation; 2, severe inflammation. Arthritis development was scored macroscopically, with a maximum possible score of 8 per mouse. Mice with a score of ≥ 6 were killed for ethical reasons. Scoring was performed blindly by two independent observers, without knowledge of the experimental groups [18].

### Lymphoid tissue preparation

Spleens were harvested from β-cat^DEL^ and control mice and homogenized in ice-cold Hanks’ balanced salt solution (HBSS, Sigma) using 100 μm cell strainers (BD Falcon, Sunnyvale, CA, USA).

For preparation of DCs, spleens were pre-incubated with 400 units collagenase type IV (Worthington, Lakewood, NJ, USA) and 0.1 units DNase I (Sigma Aldrich) in RPMI-1640 culture medium (Lonza, Basel, Switzerland) at 37°C for 40 minutes. Subsequently, 10 μM of EDTA was added to each sample and incubated for another 5 minutes.

### Flow cytometry

For intracellular cytokine staining, splenocytes were stimulated for 4 hours with phorbolmyristate acetate (PMA) (0.05 μg/ml; Sigma-Aldrich) and ionomycin (0.5 μg/ml; Sigma-Aldrich) in the presence of GolgiStop (BD Bioscience, Sunnyvale, CA, USA). Cells (3x10^6^ per staining) were stained for 30 minutes at 4°C for extracellular markers and subsequently fixed with 2% paraformaldehyde (PFA) and permeabilized in 0.5% saponin. Cells were then stained for 40 minutes at 4°C for intracellular markers. For intracellular transcription factors staining a FoxP3 staining buffer set (eBioscience, San Diego, CA, USA) was used according to the manufacturer’s instructions. The following antibodies were used: anti-CD3 (BV785), anti-TCRγδ (BV605), anti-CCR4 (APC), anti-CCR6 (PE), anti-CXCR3 (PE-Cy7), anti-IL4 (PE-Cy7), anti-IL17A (BV 421), anti-IL22 (A647), anti-IL10 (A488), anti-TNFα (PerCP-Cy5.5), anti-MHCII (A647), anti-CD80 (PerCP-Cy5.5) from Biolegend; anti-CD4 (PE-Cy7), anti-CD8α (PE-Cy5; APC-H7), anti-CD25 (PerCP-Cy5.5), anti-CD62L (APC-Cy7), anti-NK1.1 (A700), anti-CXCR5 (FITC), anti-IL17A (A647), anti-IL12 (p40/p70) (PE), anti-PD-L2 (PE) from BD Pharmingen; anti-IL17F (PE), FoxP3 (PE-Cy7), anti-CD11c (PE-Cy5.5), anti-MHCII (A700) from eBioscience; anti-CD4 (V500), anti-CD11c (BV786), anti-IFNγ (PE-CF594), anti-IL6 (V450), anti-CD40 (BV421), anti-CD86 (BV605), anti-PD-1 (BV421, PE-CF594) from BD Horizon; and anti-β-catenin (A647) from Cell Signalling. Samples were acquired on a BD LSR Fortessa (BD Biosciences, San Jose, CA, USA). Flow cytometric results were analysed using FlowJo, version 7.6.5.

### Histological analysis

Three arthritic knees from control and 3 from β-cat^DEL^ mice were fixed for 4 days in 10% formalin, decalcified in 5% formic acid and embedded in paraffin. Tissue sections (7 μm) were made and stained with hematoxylin and eosin. Sections were assessed for joint inflammation (infiltrate and exudate) and bone and cartilage erosions [19]. The severity of inflammation was scored on a scale of 0–3 (0 = no cells, 1 = mild cellularity, 2 = moderate cellularity, and 3 = maximal cellularity). The severity of bone and cartilage damage was scored separately based on bone and cartilage erosions score using a scale from 0–3 (0 = no erosion, 1 = mild erosion, 2 = moderate erosion, and 3 = maximal erosion) and on chondrocyte death (0 = no cell death, 1 = mild cell death, 2 = moderate cell death, and 3 = maximal cell death). Histopathological changes in the knee joints were scored in the patella/femur region on three semiserial sections of the joint, spaced 70 μm apart.

### Measurement of collagen-specific antibodies

For the measurement of CII-specific antibodies in serum, 96-well microtiter plates were coated overnight with 1 μg/ml chicken or mouse CII (Chondrex, Redmond, WA, USA). Serum samples and reference samples were diluted serially and incubated in the plates for 3 hours at room temperature. Secondary antibodies (goat-anti-mouse biotinylated IgG total, IgG1, and IgG2c; Southern Biotechnology) were incubated for 1 hour at room temperature. Conjugation was performed with poly-HRP-streptavidin for 30 minutes (Sanquin, Amsterdam, The Netherlands). Substrate development was done with 3,3',5,5'-Tetramethylbenzidine (TMB) (KPL, Maryland, USA). Plates were developed for approximately 15 minutes and the reaction was stopped with 0.5 M H_2_SO_4_. Optical density was measured at 450 nm.

### Quantitative real-time PCR analysis

RNA was extracted from synovium and cortex from 6 control and 6 β-cat^DEL^ naïve mice using TRIzol^®^ reagent (Life technologies) according to manufacturer’s instructions and after the final precipitation dissolved in RNase-free water. Total RNA (0.5 μg) was reverse transcribed into first-strand cDNA with Superscript II reverse transcriptase (Invitrogen) and 50 ng random primers during 50 minutes at 50°C in a total volume of 20 μl. From all samples, a 1:20 dilution was made and used for Quantitative real-time PCR (qPCR) analysis.

qPCR primers were designed with ProbeFinder software and probes were chosen from the universal probe library (Roche Applied Science, Indianapolis, IN). qPCR was performed using the ViiA 7 real-time PCR system (Life Technologies) and analysed using ViiA™ 7 software (Life Technologies). The transcription values were normalized against Glyceraldehyde 3-phosphate dehydrogenase (GAPDH) transcription. Primers and probes used: GAPDH forward 5’-AGCTTGTCATCAACGGGAAG-‘3, GAPDH reverse 5’-TTTGATGTTAGTGGGGTCTCG-‘3, probe 9; Wnt2b forward 5’-CCGGGACCACACTGTCTTT-‘3, Wnt2b reverse 5’-GCTGACGAGATAGCATAGACGA-‘3, probe 16; Wnt3 forward 5’-CTCGCTGGCTACCCAATTT-‘3, Wnt3 reverse 5’-GAGGCCAGAGATGTGTACTGC-‘3, probe 81; Wnt5a forward 5’-ACGCTTCGCTTGAATTCCT-‘3, Wnt5a reverse 5’-CCCGGGCTTAATATTCCAA-‘3, probe 55; Wnt5b forward 5’-AGCACCGTGGACAACACAT-‘3, Wnt5b reverse 5’-AAGGCAGTCTCTCGGCTACC-‘3, probe 53; Wnt7a forward 5’-CGCTGGGAGAGCGTACTG-‘3, Wnt7a reverse 5’- CGATAATCGCATAGGTGAAGG-‘3, probe 12; Wnt8a forward 5’-ACTGCGGCTGTGACGAGT-‘3, Wnt8a reverse 5’-CCCGAACTCCACGTTGTC-‘3, probe 75; Wnt8b forward 5’- GTGGACTTCGAAGCGCTAA-‘3, Wnt8b reverse 5’-GGGTGTACATAACCCCAGCA-‘3, probe 94; Wnt9a forward 5’-CGAGTGGACTTCCACAACAA-‘3, Wnt9a reverse 5’- GGCATTTGCAAGTGGTTTC-‘3, probe 19.

### Statistical analysis

Differences between groups were tested with the Mann-Whitney *U* test, unless noted otherwise. Data were analysed using Graph-Pad Prism, version 5.01 (Graphpad Software Inc., La Jolla, CA). *P* values less than 0.05 were considered significant (* p < 0.05; ** p < 0.01; *** p < 0.*001*). Error bars are presented as standard error of the mean (SEM).

## Results and Discussion

### Ablation of *β-catenin* specifically in CD11c^+^ cells did not influence the maturation and activation of BMDCs in response to TLR2 or TLR4 activation

**Fig 1:**
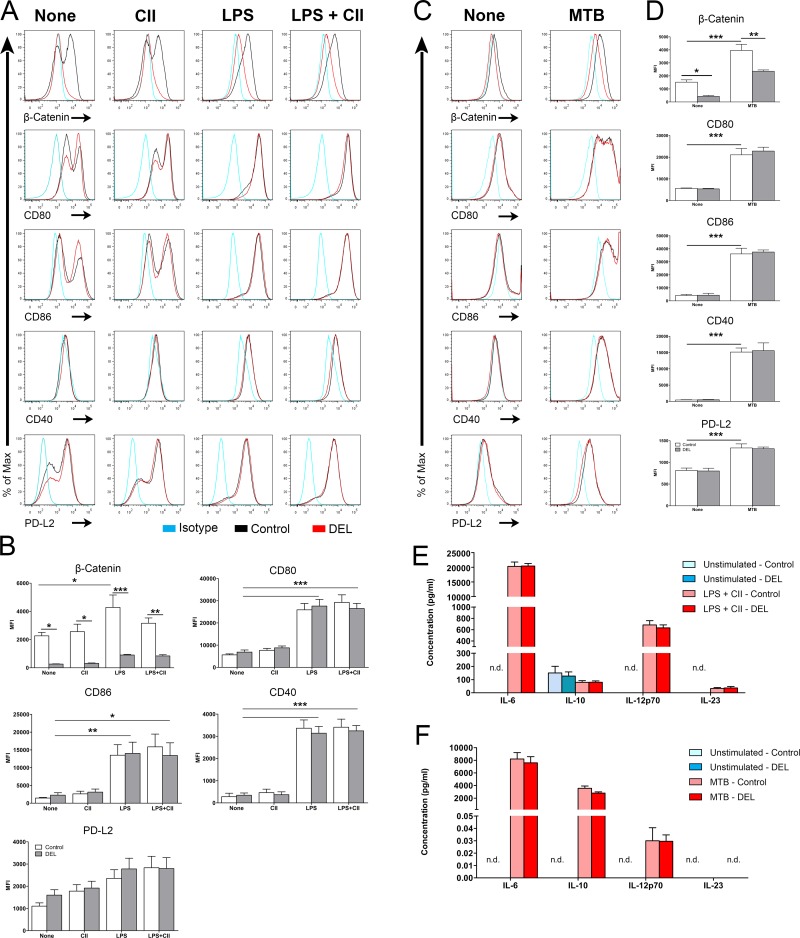
Ablation of β-catenin did not influence the maturation and activation of BMDCs. BMDCs were cultured in the presence of GM-CSF for 10 days, and either left unstimulated (spontaneous maturation), treated with LPS (1 ng/ml), or (chicken) CII (50 μg/ml) or LPS and CII (50 μg/ml; 1 ng/ml respectively) (A, B) or with *Mycobacterium tuberculosis* (MTB, 25 μg/ml) (C, D). Twenty-four hours after stimulation, BMDCs were collected and analysed by flow cytometry for their maturation status. The levels of β-catenin, co-stimulatory molecules (CD40, CD80, CD86), the co-inhibitory molecule PD-L2 were measured in CD11c^+^ BMDCs derived from control and β-cat^DEL^ mice. Levels of cytokines associated with tolerance (IL-10) and T cell skewing (IL-23, IL12p70 and IL-6) were determined by ELISA of the supernatants of control and β-cat^DEL^ BMDCs, left unstimulated or pulsed with CII and LPS (E) Or of the supernatants from BMDCs, left unstimulated or stimulated with MTB (F). All culture conditions were performed in triplicates. Data are representative of two to three independent experiments (*n* = 6–9 mice/group). Data on (B and D) were analysed by one-way ANOVA followed by a Bonferroni post-test and data on (E and F) was analysed by Mann-Whitney *U* test. Data are presented as mean ± SEM. *p< 0.05; **p<0.01; ***p<0.*001*. N.d. Not detectable.

BMDCs are a convenient source of large amounts of DCs and express high levels of β-catenin. Thus, we investigated the efficiency of Cre-mediated recombination of the β-catenin gene in BMDCs generated from β-cat^DEL^ mice by studying the level of β-catenin protein expression using flow cytometry. We observed that the mean fluorescence intensity (MFI) of β-catenin was significantly decreased by 88% in immature β-cat^DEL^ as compared to control BMDCs (Fig 1A–1D). A similar decrease was observed in DCs isolated from lungs and skin of β-cat^DEL^ mice (Ober-Blöbaum, J.L. and Clausen, B.E. *unpublished data*). Next, we asked whether BMDC activation/maturation alters β-catenin expression. Therefore, we measured the level of β-catenin in BMDCs left untreated or pulsed for 24 hours with CII (Fig 1A and 1B), a TLR4 ligand (LPS) (Fig 1A and 1B), a combination of both (LPS and CII) (Fig 1A and 1B) or with *M*. *tuberculosis* (MTB) (Fig 1C and 1D). In the control, stimulation with LPS or with MTB, but not CII, resulted in an increase in the levels of β-catenin compared to unstimulated cells (Fig 1A, 1B, 1C and 1D). As expected, in the β-cat^DEL^ group, LPS (Fig 1A and 1B) or MTB (Fig 1C and 1D) stimulation both failed to trigger any expression of β-catenin. These data indicate efficient Cre-mediated deletion of β-catenin in immature and mature β-cat^DEL^ BMDCs and that TLR2- or TLR4-induced maturation of BMDCs by MTB or LPS respectively leads to an upregulation of β-catenin, which is in line with previous studies [15].

Since Jiang *et al*. reported that the phenotypic maturation of cluster disrupted BMDCs was mediated by β-catenin translocation to the nucleus [12], we compared the expression levels of the co-stimulatory molecules CD80, CD86 and CD40 and of the co-inhibitory molecule PD-L2 on the surface of β-cat^DEL^ and control BMDCs. Treatment of BMDCs with LPS or LPS + CII (Fig 1A and 1B) or MTB (Fig 1C and 1D) induced high expression of CD80, CD86 and CD40 maturation markers, while addition of CII antigen alone elicited no effects on these molecules. Intriguingly, the phenotypic maturation of control and β-cat^DEL^ BMDCs was similar (Fig 1A, 1B, 1C and 1D). These results indicate that in contrast to the observation made by Jiang *et al*., where overexpression of *β-catenin* resulted in phenotypic DC maturation, the deletion of *β-catenin* does not impair BMDC maturation in response to a TLR2 or a TLR4 ligand [12]. Our data is consistent with previous study performed by van der Lugt *et al*. where it was demonstrated that BMDCs infected with shRNAs against *β-catenin* responded normally to LPS stimulation [20]. The same authors showed also that BMDCs depleted from β-catenin using the same strategy do not respond to mechanical stimulation.

**Fig 2:**
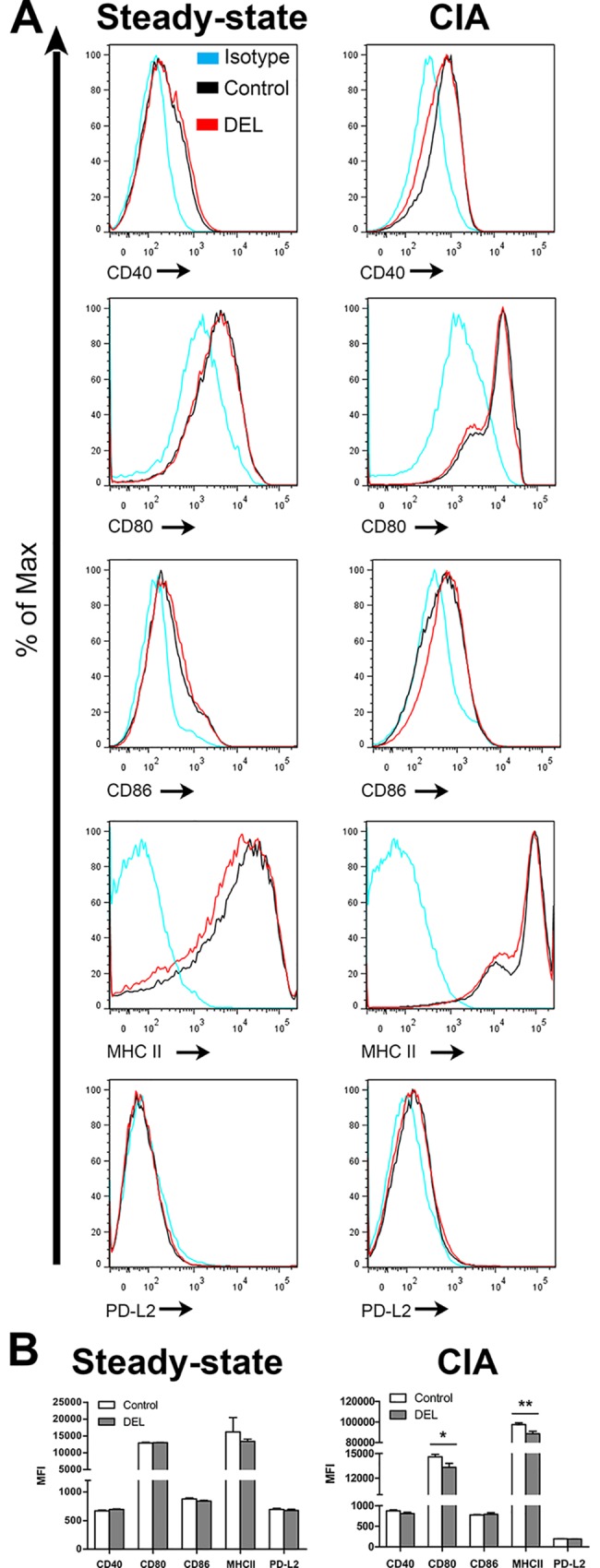
Deletion of β-catenin in DCs during CIA resulted in a reduction of the co-stimulatory molecules CD80 and MHC-II in CIA. Representative histogram of CD40, CD80, CD86, MHC-II and PD-L2 expression by CD11c^+^ DCs isolated from the spleen under steady-state condition (left column) and during CIA (right column) from control and β-cat^DEL^ mice (A). Spleen from CIA mice were collected at day 35 after initial immunization. Bar diagrams show the quantification of the MFI relative to the expression of costimulatory molecules (B). No differences in the expression of costimulatory molecules between naïve control and β-cat^DEL^ naïve mice were observed (CD40: *p = 0*.*132;* CD80: *p = 0*.*289;* CD86: *p = 0*.*132;* MHC-II: *p = 0*.*575; PD-L2*: *p = 0*.*589;* n = 6 mice per group). In the CIA group (n = 10 mice per group), differences in CD80 (*p = 0*.*028*) and MHC-II (*p = 0*.*009*) were found between control and β-cat^DEL^ mice. No differences were found in the expression of CD40 (*p = 0*.*121*), CD86 (*p = 0*.*082*) and PD-L2 (*p = 0*.*325*) in the CIA group. Data are presented as mean + SEM. Asterisks indicate a significant difference compared with the control (Mann-Whitney *U* test). *p< 0.05; **p<0.*01*.

In line with the unaltered immature phenotype of untreated β-cat^DEL^ BMDCs, we did not observe any differences in the expression of co-stimulatory and co-inhibitory molecules on splenic CD11c^+^ DCs from naïve β-cat^DEL^ and control mice (Fig 2A and 2B, left panel).

Manicassamy *et al*. demonstrated that DCs isolated from the lamina propria of β-cat^DEL^ mice expressed higher mRNA levels of the pro-inflammatory cytokines IL-23 and IL-6 and lower mRNA levels of the anti-inflammatory cytokines IL-10 [13]. Therefore, we examined whether the lack of *β-catenin* in BMDCs changed the expression of signature cytokines for T helper cell skewing. The levels of IL-6, IL-10, IL-12p70 and IL-23 were measured in the supernatant of cells left unstimulated or pulsed with LPS and CII. Except for the anti-inflammatory IL-10, unstimulated cells fail to produce any of the pro-inflammatory cytokines tested (Fig 1E). As expected, stimulation with LPS and CII resulted in a marked induction of IL-6, IL-12p70 and IL-23 but not of IL-10 (Fig 1E). No differences were observed in cytokine levels between control and β-cat^DEL^ BMDCs in any of the conditions tested (unstimulated; LPS and CII) (Fig 1E).

Our data obtained using BMDCs revealed that deletion of *β-catenin* did not affect the spontaneous or TLR4-induced DC maturation and cytokine production *in vitro*. The specific LPS-mediated signalling cascade is initiated by the interaction between LPS and TLR4, adaptor proteins, including myeloid differentiation protein 88 (MyD88), tumour necrosis factor receptor-associated factor 6 (TRAF6), and MyD88 adapter-like (MAL), are subsequently recruited β-catenin. Finally, downstream signalling pathways such as MAPKs and NF-kB are activated [21]. Thus, our data suggest that LPS-mediated signalling and LPS-mediated maturation is independent of β-catenin. By contrast, others demonstrated that β-catenin is necessary for BMDC maturation and tolerogenic DC activation by mechanical stimulation [12]. These findings suggested that the release of the β-catenin pool associate with E-cadherin, at the adherens junctions, together with disruption of cell-cell contact might boost the levels of the free-β-catenin in the cytosol and consequent increase in wnt signalling resulting in maturation of the BMDCs. Moreover, these two processes, LPS and mechanic stimulation, seem to be independent once treatment of BMDCs with anti-E-cadherin did not inhibit maturation induced by LPS and DCs lacking *Tlr4*, that cannot respond to LPS, exhibited robust maturation after mechanical stimulation [12].

Recently, it has been shown that stimulation of BMDCs lacking LRP5/6, and consequently β-catenin signalling, with *M*. *tuberculosis* resulted in an increase of IL-6, TNFα, IL-12 and a reduction of IL-10 [14]. We stimulated BMDCs with *M*. *tuberculosis* and in contrast to the observations performed by Suryawanshi *et al*. we did not observed differences in the levels of IL-6, IL-10 and IL-12p70 between control and β-cat^DEL^ BMDCs (Fig 1F). Suggesting that upstream signals of the wnt signalling are important to control the expression of the differences cytokines, rather than β-catenin alone.

Also, other studies demonstrated that augmented *β-catenin* signalling leads to enhanced phenotypic DC maturation without production or secretion of immunostimulatory cytokines [12, 15, 20, 22]. Moreover, treatment of immature DCs with a GSK-3β inhibitor, which led to an increase in β-catenin signalling, resulted in an increase in the expression of CD80, CD86 and CD40 on the cell surface, *in vitro* [23].

In summary, deletion of *β-catenin* from BMDCs did not influence the expression levels of co-stimulatory molecules and cytokine production.

### Lack of *β-catenin* specifically in CD11c^+^ DCs did not affect T cell homeostasis in the steady state

**Fig 3:**
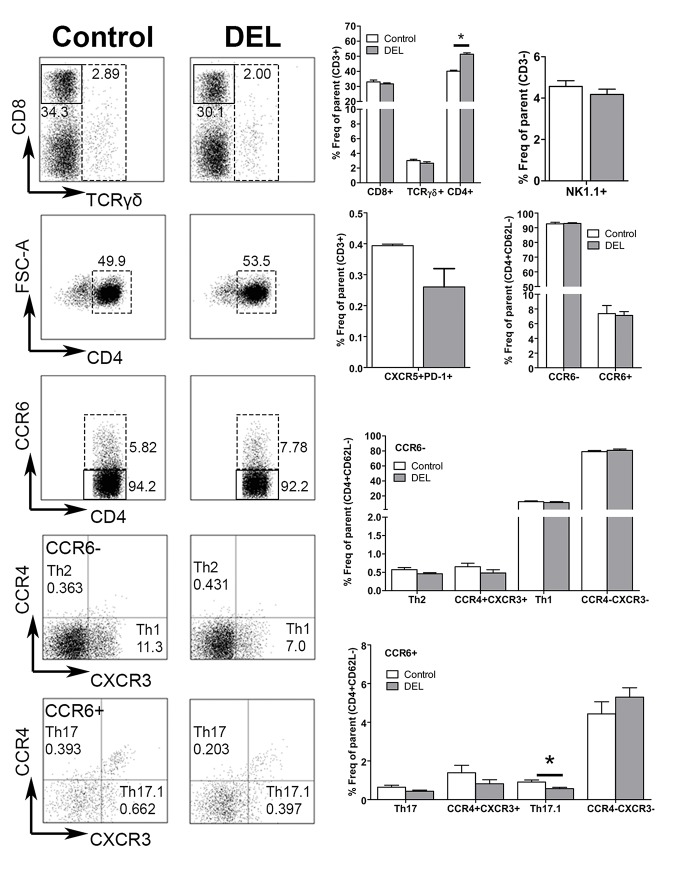
Ablation of β-catenin in DCs resulted in elevated total CD4^+^ T-cell numbers under steady state condition. The left panels depict representative FACS plots of CD8^+^, TCRγδ^+^, CD4^+^, CD4^+^CCR6^-^ and CD4^+^CCR6^+^, Th17 or Th17.1 and Th2 or Th1 T cells isolated from the spleens of control and β-cat^DEL^ mice under steady state condition. The right panels illustrate the quantification of the percentages of CD8^+^, TCRγδ^+^, CD4^+^, Tfh (CXCR5^+^PD-1^+^), CD4^+^CCR6^+^ and CD4^+^CCR6^-^ T cells, expressed in frequency of total T cells (CD3^+^); the frequency of NK T cells (NK1.1^+^) is expressed in percentage of CD3^-^ cells. The percentages of Th cells were expressed in frequency of effector T-cells (CD3^+^CD4^+^CD62L^-^). N = 6 mice per group. Data are presented as mean + SEM. Asterisks indicate a significant difference compared with the control (Mann-Whitney *U* test). *p< 0.*05*.

**Fig 4:**
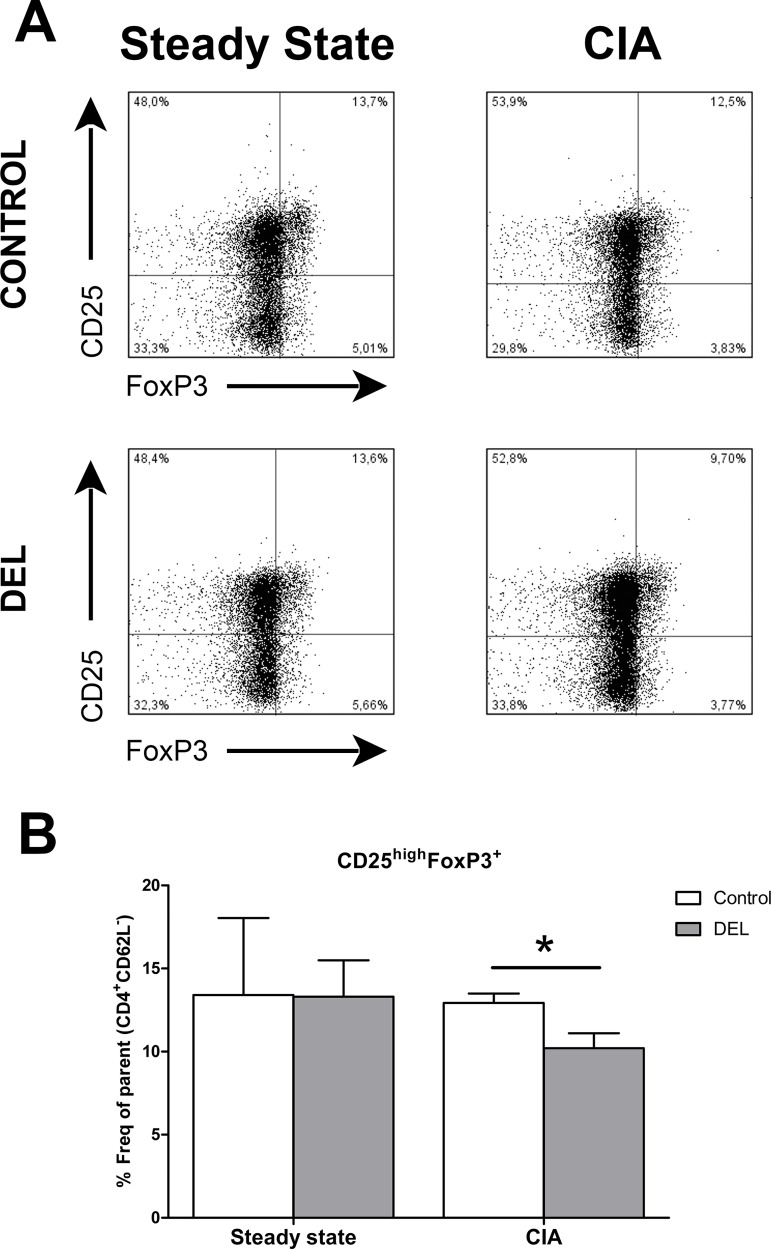
Ablation of β-catenin in DCs downregulates Tregs during CIA but not under steady state condition. Representative FACS plots of Tregs (CD4^+^CD62L^-^CD25^high^FoxP3^+^) isolated from splenocytes from control and β-cat^DEL^ mice in the steady state and subjected to CIA (35 days after the first immunization) (A). Bar diagrams depicting the frequency of Tregs, expressed as the percentage of effector T cells (CD4^+^CD62L^-^), in control and β-cat^DEL^ groups in the steady state (n = 6 mice per group, left) and in mice subjected to CIA (n = 10 mice per group, right) (B). While in the steady state no differences in the percentage of Tregs were found between the two groups (control = 13.41% ± 4.624 versus β-cat^DEL^ = 13.31% ± 2.188, *p = 0*.*9372)*, in the CIA mice we observed a reduction of 20% in the β-cat^DEL^ compared to the control group (control = 12.93% ± 0.5691 versus β-cat^DEL^ = 10.21% ± 0.9003, *p = 0*.*0435)*. Data are presented as mean + SEM. Asterisks indicate a significant difference compared with the control (Mann-Whitney *U* test). *p< 0.*05*.

To determine whether ablation of *β-catenin in* DCs has an indirect effect on the T cell compartment in naïve animals, we studied the homeostasis of CD8^+^, TCRγδ^+^, total CD4^+^ and NK1.1^+^ (natural killer, NK) T cells. An increase in the frequency of total CD3^+^CD4^+^ T cells was observed in β-cat^DEL^ as compared to control mice (control = 48.08% ± 0.611 versus β-cat^DEL^ = 51.30% ± 0.942, *p = 0*.*0304*). No changes in the frequencies of CD3^+^CD8^+^, CD3^+^TCRγδ^+^ and NK T cells were detected (Fig 3). The percentage of T follicular helper (Tfh) cells (CD3^+^CD4^+^CD62L^-^CXCR5^+^PD-1^+^) was comparable between the two groups (Fig 3). To further assess the frequencies of different Th cell subsets, such as Th1, Th2, Th17 and Th17.1 (also called non-classic Th1 cells having characteristics of both Th17 and Th1 cells), we developed a flow cytometry gating strategy to identify these T cell populations based on their unique combination of chemokine receptors (CCR6, CCR4 and CXCR3) present on their cell surface (S1 Fig). First, CD4^+^ T cells were divided into two populations according to their CCR6 expression. In the CCR6^+^ compartment, Th17 cells were defined as CCR4^+^CXCR3^-^ and Th17.1 as CCR4^-^CXCR3^+^ cells. In the CCR6^-^ compartment, Th2 cells were defined as CCR4^+^CXCR3^-^ and Th1 as CCR4^-^CXCR3^+^ cells (S1 Fig) [24, 25]. No differences were observed between the percentages of CD4^+^CD62L^-^CCR6^-^ (control = 7.320% ± 1.1060 versus β-cat^DEL^ = 7.043% ± 0.5201, *p = 0*.*5887*) and CD4^+^CD62L^-^CCR6^+^ (control = 0.7185% ± 0.5215 versus β-cat^DEL^ = 0.5203% ± 0.1027, *p = 0*.*1797*) T cell populations in spleens of naïve β-cat^DEL^ and control mice (Fig 3). The percentage of Th17.1 cells was reduced in the β-cat^DEL^ compared to the control group (control = 0.9088% ± 0.1058 versus β-cat^DEL^ = 0.5648% ± 0.4809, expressed in percentage of effector T cells, *p = 0*.*0411*) (Fig 3). No further changes were detected for any other Th cell subsets within both the CCR6^+^ and CCR6^-^ compartment (Fig 3), including similar levels of splenic Tregs (CD4^+^CD25^high^FoxP3^+^) between the β-cat^DEL^ and control group (control = 13.41% ± 4.624 versus β-cat^DEL^ = 13.31% ± 2.188, expressed in percentage of effector T cells, *p = 0*.*9372*) (Fig 4A, left panel).

**Fig 5:**
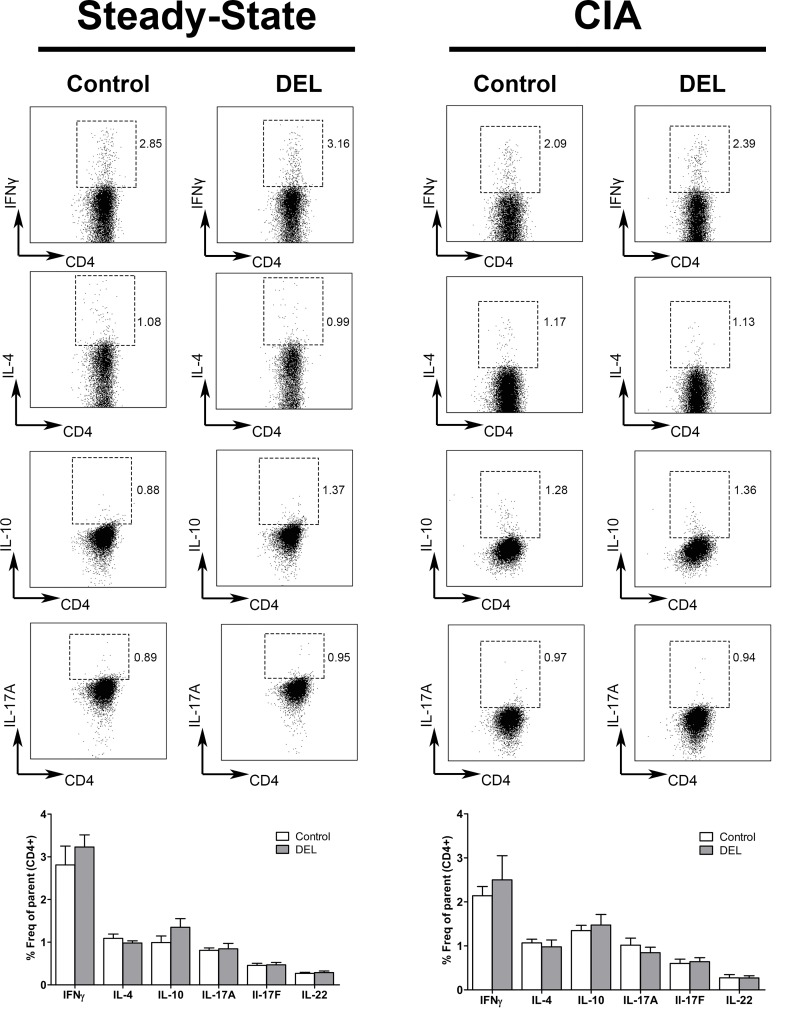
β-catenin deletion in DCs did not affect the cytokine production by CD4^+^ T cells in the steady state or during CIA. Representative FACS plots illustrating the pro-inflammatory cytokines IFNγ, IL-4, IL-17A and anti-inflammatory IL-10 produced by CD4^+^ T cells isolated from spleens of steady-state (left panel, n = 8 mice per group) and CIA (right panel, n = 10 mice per group) mice, on day 35 after the first immunization. Cells were stimulated for 4 hours with phorbolmyristate acetate (PMA) (0.05 μg/ml) and ionomycin (0.5 μg/ml) in the presence of GolgiStop. The frequencies of the different cytokine-secreting Th cells were similar in the β-cat^DEL^ and control groups in both conditions. Data are presented as mean ± SEM.

Though we did not observe changes in the homeostasis of the different T cell subsets in the steady state, their cytokine production could be affected during inflammation. Therefore, we studied whether deletion of *β-catenin* in DCs influenced the cytokine production by the different T cell subsets. Splenocytes were isolated from naïve β-cat^DEL^ and control mice and stimulated *in vitro* with phorbolmyristate acetate (PMA) and ionomycin for 4 hours. We did not find differences in the production of IFNγ, IL-4, IL-10, IL-17A, IL-17F and IL-22 by CD4^+^ T cells (Fig 5, left panel) as well as in the production of IFNγ by CD3^+^CD8^+^ and CD3^-^NK1.1^+^ T cells (data not shown). Although, we observed a decrease in the Th17.1 subpopulation in β-cat^DEL^ mice, we did not observe a decrease in the levels of the IL-17 and IFNγ cytokines secreted by Th17.1 cells. One explanation could be that the cytokine levels were analysed in the bulk CD4^+^ T cells and not specifically in each T cell subset. Since once the Th17.1 cells are approximately 1.50–2.5% of all the CD4^+^ T cells we might not be able to distinguish the contribution of this particular population in the production of these cytokines that are also produced by Th1 (IFNγ) and Th17 (IL-17).

In summary, deletion of *β-catenin* in CD11c^+^ DCs did not indirectly affect splenic T cell populations under steady state conditions, which is in line with previous studies demonstrating that lack of *β-catenin* specifically in DCs did not change the percentages of splenic Tregs, Th1 or Th17 cells [13, 15]. In contrast, in the intestine ablation of *β-catenin* in CD11c^+^ cells resulted in a decrease of Tregs and an increase of Th17 and Th1 cells [13]. Manicassamy *et al*. suggested that the difference in Treg frequencies observed between tissues, i.e. the spleen and intestine, might be due to the expression levels of wnt ligands and frizzled receptors by *CD11c* ^*+*^ cells. They reported that CD11c^+^ cells isolated from the lamina propria of control mice expressed higher amounts of *Wisp1* and *Axin1* mRNA than CD11c^+^ cells isolated from the spleen. This resulted in higher activation of wnt signalling in intestinal CD11c^+^ cells programming them to induce Tregs [13].

### Deletion of *β-catenin* in CD11c^+^ cells did not alter the course nor the severity of autoimmune collagen-induced arthritis (CIA)

**Fig 6:**
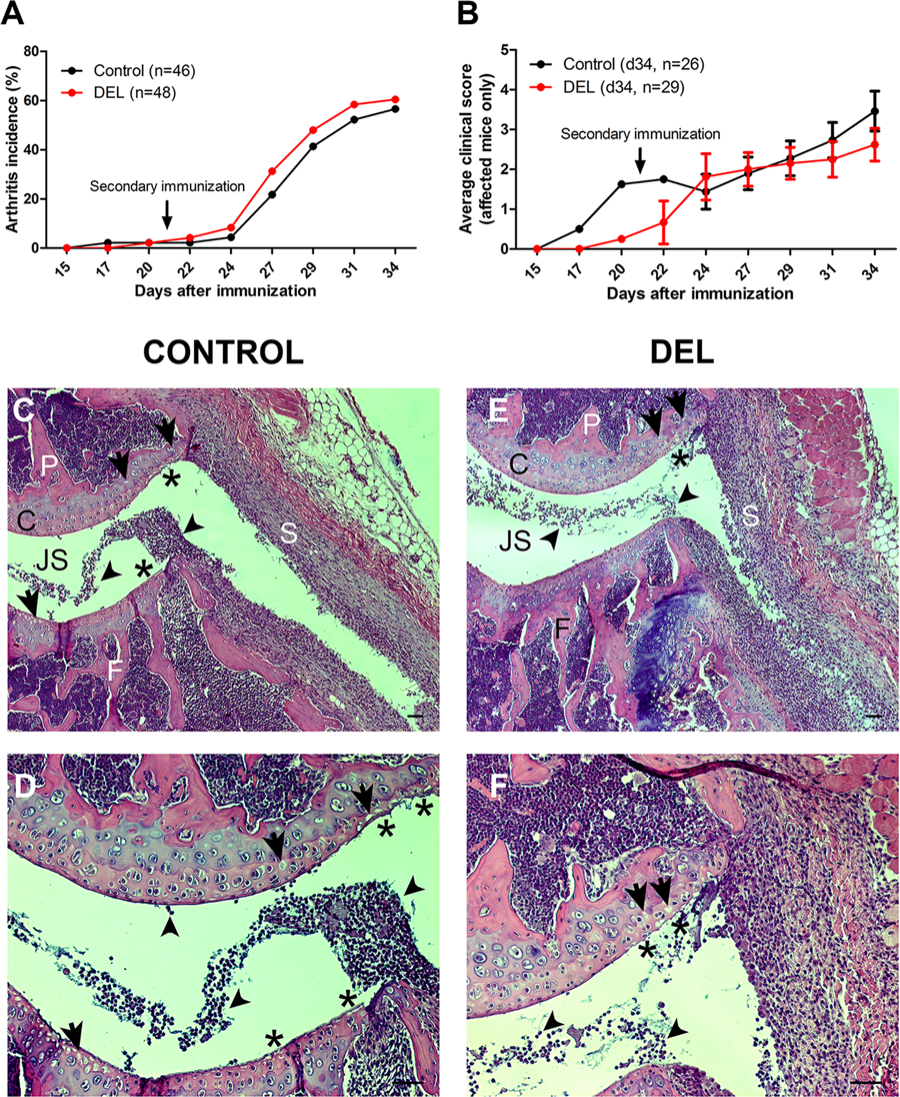
Deletion of β-catenin in DCs did not alter the onset and severity of autoimmune CIA. Arthritis induction could be observed in mice as early as 17 days after immunization (A). There was no difference in the CIA incidence between the control (arthritis incidence = 56.5%, n = 46) and β-cat^DEL^ (arthritis incidence = 60.4%, n = 48) group (A). In addition, no difference in the disease severity was observed between the control (n = 26) and β-cat^DEL^ group (n = 29) (B). The disease severity reached a maximal average score of 3.5 ± 0.5 in the control and 2.6 ± 0.4 in the β-cat^DEL^ group on day 34 (*p = 0*.*3765*) (B). Representative histological images of the knees of CIA control (C and D) and β-cat^DEL^ (E and F) mice collected on day 35 after the first immunization. The tissue sections were stained with hematoxylin and eosin. Scale bar: 50 μm. JS = joint space; P = patella; S = synovial membrane; F = femur; C = cartilage.

Although the lack of β-catenin in CD11c^+^ cells did not significantly affect the immunological steady state, it may exacerbate a DC/T cell-mediated autoimmune disease model. Therefore, we subjected β-cat^DEL^ and control mice to autoimmune CIA. Arthritis induction could be observed in mice as early as 17 days after the first immunization. The CIA incidence in our experiments (56–60%) is in line with previous reports using mice on a C57/BL6 genetic background [26–28] (Fig 6A). The overall CIA incidence, on day 34 after the first immunization, was very similar between the control (arthritis incidence = 56.5%, n = 46) and β-cat^DEL^ (arthritis incidence = 60.4%, n = 48) mice (Fig 6A). Moreover, the onset and progression of the disease was also similar in both groups (Fig 6A).

In mice that developed CIA, the disease severity reached an average score of 3.5 ± 0.5 in the control (n = 26) and 2.6 ± 0.4 in the β-cat^DEL^ (n = 29) group on day 34 (*p = 0*.*3765*) (Fig 6B). Statistical differences between the two groups were not reached at any of the time points analysed (day 27, *p = 0*.*8560*; day 29, *p = 0*.*5304*; day 31, *p = 0*.*2539*). Histopathological analysis of knee joints of control and β-cat^DEL^ mice showed a similar extent of synovial inflammation with high numbers of cells infiltrating into the synovial tissue (control = 1.123 ± 0.228 versus β-cat^DEL^ = 1.087 ± 0.007, *p = 1*.*000*) (Fig 6C–6F, arrowheads). Mild destruction of cartilage in the patellar and femoral regions, measured as the degree of chondrocyte death (control = 1.116 ± 0.089 versus β-cat^DEL^ = 0.650 ± 0.143, *p = 0*.*100*) (Fig 6C–6F, arrows) and cartilage surface erosion (control = 1.610 ± 0.1268 versus β-cat^DEL^ = 0.980 ± 0.175, *p* = *0*.*100*) (Fig 6C–6F, asterisks), was observed in both groups. Bone erosion was detected sporadically without differences between both groups.

In summary, deficiency of *β-catenin* in DCs did not accelerate the onset, progression or exacerbate the severity of CIA.

The impact of *β-catenin* deficiency in DCs was studied before in a mouse model of inflammatory bowel disease (IBD) induced by dextran sodium sulphate (DSS), and for multiple sclerosis, the experimental autoimmune encephalomyelitis (EAE) [13–15]. In both models, mice lacking *β-catenin* in CD11c^+^ cells exhibited an exacerbation of the disease severity and an increase in local inflammation [13–15]. On the other hand, immunization with myelin oligodendrocyte glycoprotein peptide-pulsed DCs that were matured by mechanical disruption of E-cadherin-mediated cell adhesion leading to the activation of β-catenin signalling, could protect recipient mice against the neuropathology associated with EAE [12]. Moreover, pharmacological activation of canonical wnt/β-catenin signalling delayed EAE onset and diminished central nervous system pathology [14]. In contrast, data from this study indicate that the *β-catenin* pathway in DCs is not critical in controlling autoimmune arthritis. In order to exclude that the absence of a phenotype in our CIA model is due to a difference in the microbiota and/or housing conditions and/or in the genetic background we submitted our β-cat^DEL^ to a DSS-induced colitis model. Indeed our β-cat^DEL^ mice were more susceptible to colitis than the control animals (data not shown) which is in line with published data [13]. Together, these data suggest that the differences in phenotype in β-cat^DEL^ mice are indeed relative to the disease model.

### Lack of *β-catenin* did not influence the development of auto-antibodies in CIA

**Fig 7:**
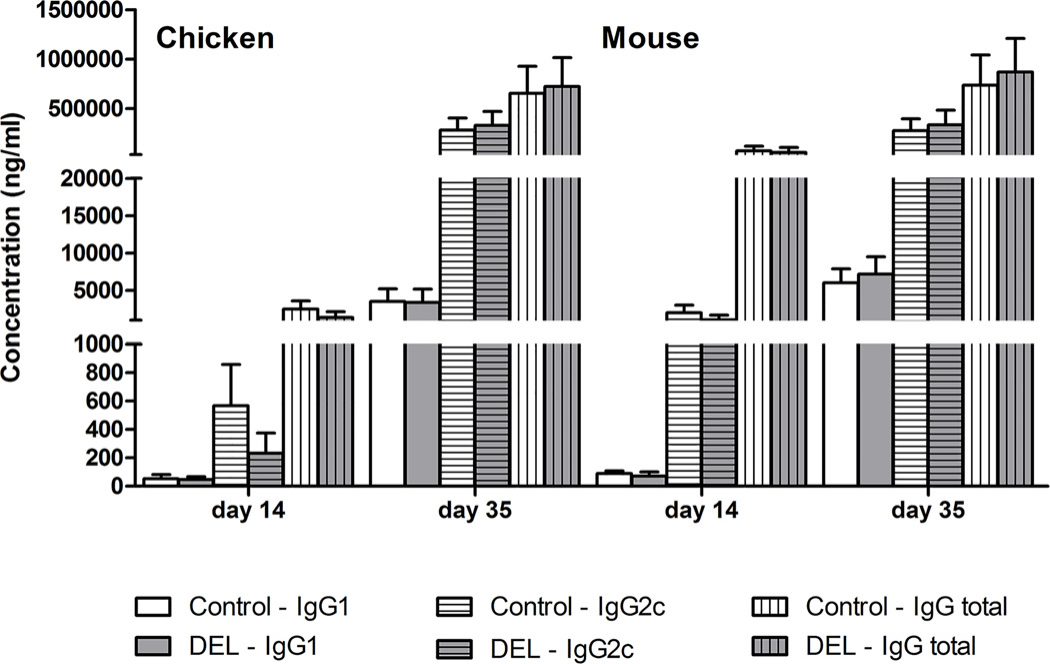
Serum levels of CII-specific antibodies in CIA mice. Enzyme-linked immunosorbent assay (ELISA) of chicken and mouse CII-specific IgG1, IgG2c, or total IgG in the serum of β-cat^DEL^ mice and control littermates at days 14 and 35 after initial immunization (n = 10 mice per group). Data are presented as mean + SEM.

Both T cell and humoral immune responses have been described to be essential in the pathogenesis of CIA. Mice immunized with CII developed elevated serum IgG1 and IgG2a/2c antibodies against CII (Fig 7), which represent a read out of systemic immune activation after immunization with chicken CII in CFA. Due to a not yet fully elucidated mechanism this immune activation will lead to specific (auto-)antibody induction against autologous mouse CII, probably directed at conserved amino acid sequences of CII, that triggers the autoimmune process. Therefore, we determined the serum levels of the specific immunoglobulins IgG1 and IgG2a/2c against both the heterologous (chicken) and autologous (mouse) CII, in order to accurately correlate auto-antibody levels with the development and severity of autoimmune arthritis. In particular, IgG2c can be found in C67BL/6 mice with CIA and is considered to play an important role in the pathogenesis of autoimmune arthritis via complement activation and immune complex mediated Fcγ receptor activation [28–30]. Circulating levels of CII-specific IgG1, IgG2c and total IgG were not detectable in sera of mice before immunization (data not shown). The Cll-specific antibody response of mice from both groups on days 14 and 35 after the first immunization was similar in magnitude and IgG subclass distribution (IgG1, IgG2c, and total IgG) (Fig 7). These data indicate that deletion of *β-catenin* in DCs did not affect the production of CII-specific antibodies by B-cells during CIA.

### Deletion of *β-catenin* in DCs resulted in decreased Tregs in CIA

**Fig 8:**
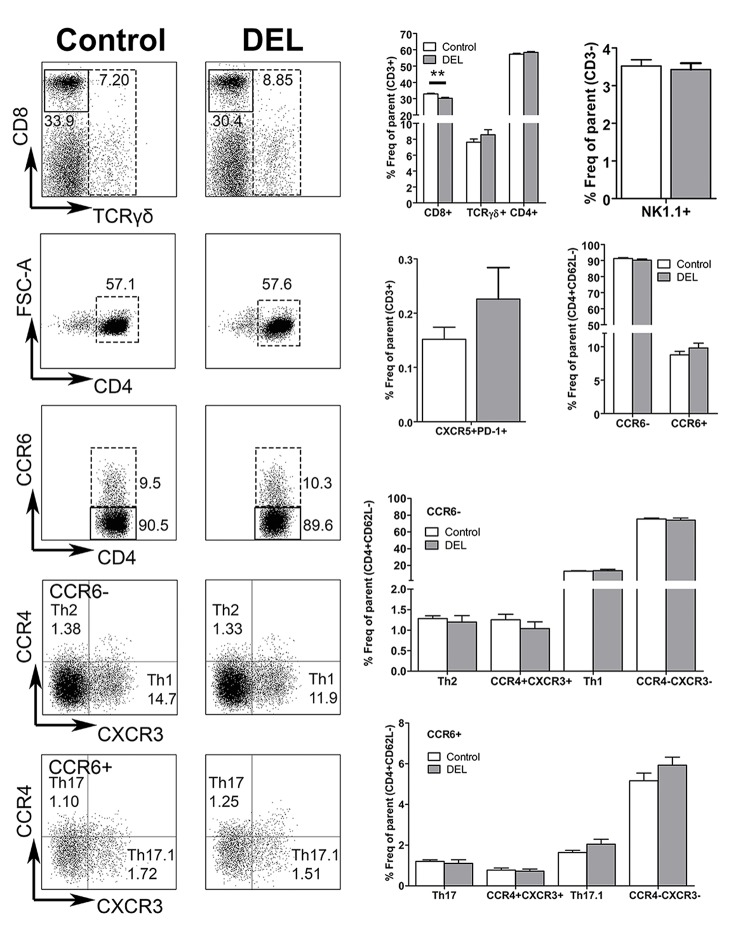
DC-specific ablation of β-catenin modulated the percentage of CD4^+^ T cell subsets in CIA mice. Left panels: Representative FACS plots depicting CD8^+^, TCRγδ^+^, CD4^+^, CD4^+^ CCR6^-^ and CD4^+^ CCR6^+^, Th17 or Th17.1 and Th2 or Th1 T cells isolated from the spleens of CIA control and β-cat^DEL^ mice at day 35 after initial immunization. Right panels: Quantification of the percentages of CD8^+^, TCRγδ^+^, CD4^+^, Tfh(CXCR5^+^PD-1^+^) and CD4^+^CCR6^+^ and CD4^+^CCR6^-^ T cells, expressed as frequency of total T cells (CD3^+^); NK T cells (NK1.1^+^) are plotted as percentage of CD3^-^ cells. The percentages of Th cells were expressed as frequency of effector T cells (CD3^+^CD4^+^CD62L^-^). N = 10 mice per group. Data are presented as mean ± SEM. Asterisks indicate a significant difference compared with the control (Mann-Whitney *U* test). *p< 0.05; **p<0.*01*.

Next, we investigated the effect of *β-catenin* ablation specifically in DCs on the T cell profile in the autoimmune CIA model. Splenocytes were isolated from CIA mice on day 35 after the first immunization. Spleens from β-cat^DEL^ CIA mice displayed similar percentages of TCRγδ^+^, total CD4^+^ and NK1.1^+^ NK T cells as compared to controls, whereas a small decrease of CD3^+^CD8^+^ T cells (control = 32.94% ± 0.450 versus β-cat^DEL^ = 30.26% ± 0.544, *p = 0*.*0036*, n = 10 mice per group) was noted (Fig 8). The percentage of Tfh cells between the two groups was comparable (Fig 8). We further assessed the frequencies of the different Th cell subsets, namely Th1, Th2, Th17 and Th17.1, as identified by differential expression of the chemokine receptors CCR6, CCR4 and CXCR3. The percentages of all these subsets were similar between both groups (Fig 8). Subsequently, we analysed whether the levels of Tregs were affected by deletion of *β-catenin* in DCs during CIA and observed a decrease of about 20% in the percentage of splenic Tregs (CD4^+^CD25^high^FoxP3^+^) in the β-cat^DEL^ group (control = 12.93% ± 0.5691 versus β-cat^DEL^ = 10.21% ± 0.9003, expressed in percentage of effector T-cells, *p = 0*.*0435*) (Fig 4A, right panel). Of note, this differs from mice under steady state conditions where the percentages of Tregs between β-cat^DEL^ and control animals were similar (Fig 4B). Kelchtermans *et al*. demonstrated that Tregs (CD4^+^CD25^+^ cells) play an important role in CIA, and that depletion of CD25^+^ cells after immunisation with CII significantly accelerated the onset of arthritis and increased the severity of CIA [29]. In agreement, others reported that Treg cell transfer markedly slowed CIA progression [30] and, more recently that induced Tregs are able to suppress the development of CIA [31].

The 20% reduction of Tregs in the absence of accelerated disease observed in this study suggests that there may be other cell types with regulatory capacity that could compensate for a decrease in Tregs. One candidate are CD4^+^FoxP3^-^ type 1 regulatory T (Tr1) cells that secrete IL-10. However, IL-10 production by CD4^+^ T cells was similar between β-cat^DEL^ and control mice (Fig 5, right panel), indicating that Tr1 cells do not compensate for the loss of Tregs in CIA. Other candidates are endogenous IL-10 secreting B cells (so called regulatory B cells). Carter *el al*. described that mice lacking regulatory B cells developed exacerbated CIA associated with a marked increase in inflammatory Th1 and Th17 cells [32]. We did not analyse the levels of regulatory B cells and further studies are needed to examine whether this cell type compensates for the reduction of Tregs, explaining why we did not observe exacerbated CIA in the β-cat^DEL^ mice.

Previous studies demonstrated that DC-specific deletion of *β-catenin* lowers Treg frequencies in tumours [33] and in the intestine during IBD, but not in the spleen nor in the central nervous system during EAE [13–15]. TLR2-mediated activation of the *β-catenin* pathway resulted in an increase in the levels of Tregs in spleens of wild type, which was attenuated in β-cat^DEL^ mice [15].

In summary, ablation of β*-catenin* specifically in DCs resulted in a decrease in CD3^+^CD8^+^ T cells and Tregs in the autoimmune CIA model.

### Deletion of *β-catenin* in DCs did not influence cytokine secretion of CD4^+^ T cells in CIA

We evaluated if deletion of *β-catenin* in DCs affected the cytokine production of CD4^+^ T cells. Therefore, the levels of pro-inflammatory cytokines IFNγ, IL-4, IL-17A, IL-17F and IL-22 and anti-inflammatory IL-10 were measured by flow cytometry in splenocytes of CIA mice on day 35 after the first immunization. The levels of the different cytokines, produced by CD4^+^ T cells, were similar between the control and β-cat^DEL^ group (Fig 5, right panel). Also, no differences in the production of IFNγ by CD8^+^ T cells (control = 2.896% ± 0.400 versus β-cat^DEL^ = 4.027% ± 1.085, *p = 0*.*9698*) and NK1.1^+^ T cells (control = 11.2% ± 1.774 versus β-cat^DEL^ = 7.83% ± 1.521, *p = 0*.*2729*) were found between the two groups. Others have shown that CD4^+^ T cells isolated from the intestines of β-cat^DEL^ mice, subjected to IBD, showed elevated mRNA expression of the Th17 cell-associated cytokines IL-17 and IL-21 and of the Th1 cytokine IFNγ as compared to control mice [13]. Similarly, ablation of *β-catenin* signalling resulted in an increase in the percentage of IFNγ^+^, IL-17^+^ and TNFα^+^ CD4^+^ T cells in the central nervous system in an EAE model [14].

In summary, deletion of *β-catenin* in DCs did not influence cytokine secretion (IFNγ, IL-4, IL-17A, IL-17F, IL-22 IL-10) of CD4^+^ T cells in CIA.

### Deletion of *β-catenin* in DCs resulted in a reduction of the co-stimulatory molecules CD80 and MHC-II in CIA

We also studied the effect of *β-catenin* deletion in DCs on the levels of co-stimulatory (CD40, CD80, CD86, MHC-II) and co-inhibitory (PD-L2) molecules on splenic CD11c^+^ cells in the context of CIA. In contrast to their comparable expression under steady state conditions, in CIA mice the levels of CD80 (*p = 0*.*0282*) and MHC-II (*p = 0*.*0089*) were significantly reduced in β-cat^DEL^ mice (Fig 2). No differences were found in the levels of CD40, CD86 and PD-L2 (Fig 2, right panel). A recent study demonstrated that CD11c^+^ DCs from β-cat^DEL^ mice isolated from tumour-draining lymph nodes expressed enhanced levels of the activation markers CD80 and CD86, and less of the co-inhibitory molecules PD-L1 and PD-L2 [33, 34]. However, no differences were found in the amount of MHC-II, CD40, CD80 and CD86 on DCs lacking LRP5/6 as compared to wild type cells upon complete Freund’s adjuvant immunization *in vivo* [14]. In contrast, Jiang *et al*. showed that overexpression of *β-catenin* increased the percentage of CD86^hi^ DCs [12].

The discrepancy of these data suggests that the β-catenin pathway regulates the activation of DCs in a context-dependent manner.

### Differential expression of wnt ligands in synovium and cortex during steady state

The differences observed in the disease models EAE, IBD and CIA might be explained by the levels of wnt ligand expression in the respective target tissues, such as brain, lamina propria and joints (bone/cartilage/synovium), as well as in the tissue-resident DCs. Suryawanshi *et al*. observed elevated *Wnt3a* and *Wnt5a* expression in the draining lymph node during disease induction and in the central nervous system during the EAE effector phase [14]. Others reported that CD11c^+^ cells isolated from the lamina propria expressed higher amounts of the wnt target genes *Wisp1* and *Axin1* than CD11c^+^ cells isolated from the spleen [35].

**Fig 9:**
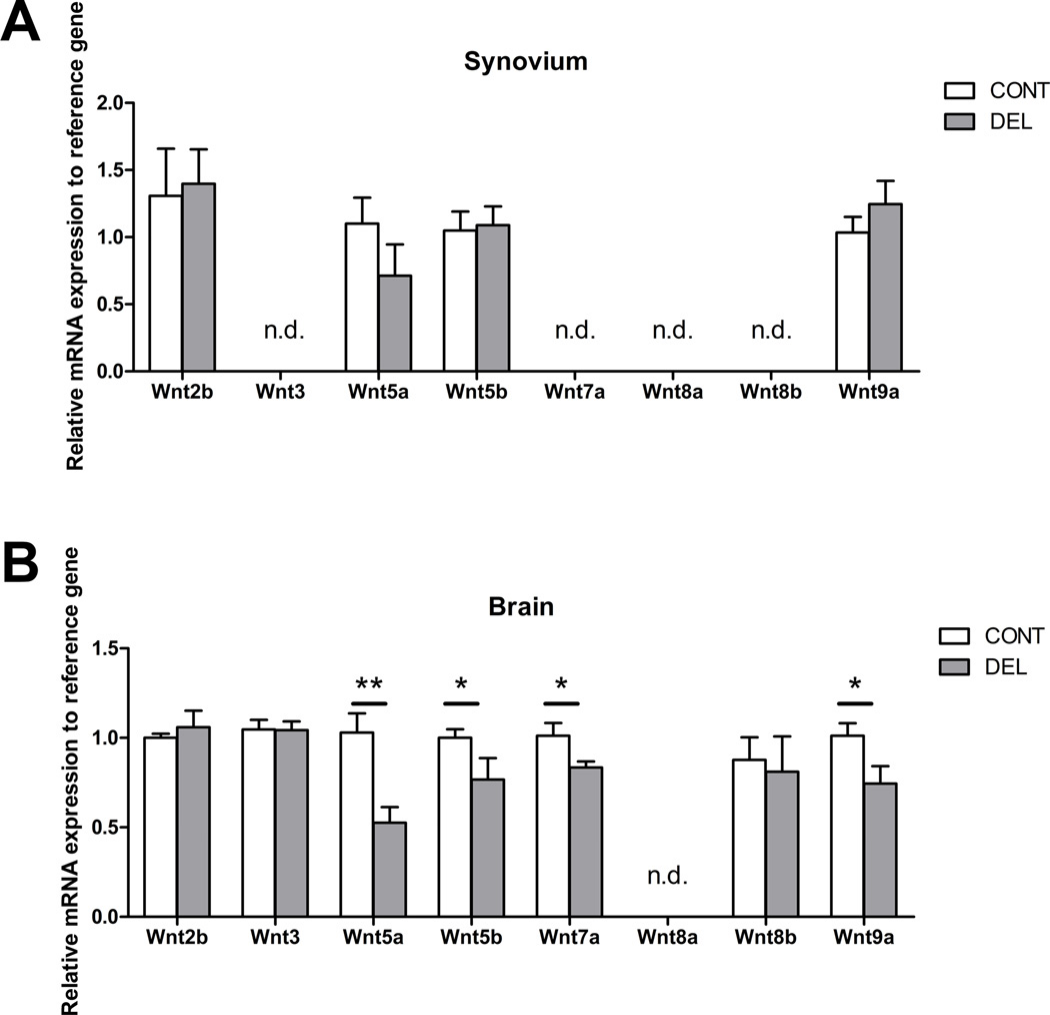
Differential expression of wnt ligands in the synovium and cortex. Transcript levels measured by quantitative real time PCR in the synovial (A) and cortical (B) tissues of control and β-cat^DEL^ mice under steady state condition. Wnt2b, wnt5a, wnt5b and wnt9a were expressed in both tissues, while Wnt3, wnt7a, wnt8b were expressed in the cortical but not in synovial tissue. The expression of wnt5a (*p = 0*.*0044*), wnt5b (*p = 0*.*0260*), wnt7a (*p = 0*.*0488)* and wnt9a (*p = 0*.*0411*) was lower in cortices of β-cat^DEL^ compared to control mice. N = 6 mice per group. Data are presented as mean ± SEM. Asterisks indicate a significant difference compared with the control (Mann-Whitney *U* test). *p< 0.05; **p<0.*01*. N.d. Not detectable.

We studied the presence of several wnt ligands, namely: wnt2b, wnt3, wnt5a, wnt5b, wnt7a, wnt8a, wnt8b and wnt9a in the synovial tissue of naïve mice. From all the wnt ligands analysed in the synovium, we only detected expression of wnt2b, wnt5a, wnt5b and wnt9a (Fig 9A). In the synovial tissue, no differences were found between the expression of wnt2b, wnt5a, wnt5b and wnt9a between control and β-cat^DEL^ mice (Fig 9A).

In human RA synovial tissue the expression levels of *Wnt1*, *Wnt5a* and *Fz5*, *Wnt7b* [36, 37] and of the wnt target gene *Wisp2* [38] were higher when compared with healthy adult tissue. The same was true for *Wnt10a* in human cartilage [36, 37].

Thus, we hypothesize that the dissimilar consequences of deletion of *β-catenin* in CD11c^+^ for the development of IBD, EAE and CIA might at least in part be explained by the local tissue expression of wnt proteins and the DC-intrinsic gene machinery that lead to differential activation of wnt target genes in DCs present in each target tissue. To support this hypothesis we analyzed the expression of the wnt ligands not only in the synovium but also in the cortex. In the cortex beside the presence of wnt2b, wnt5a, wnt5b and wnt9a, expressed in the synovial tissue, wnt3, wnt7a, wnt8b were also expressed (Fig 9B) suggesting that the wnt signalling is more prominent in this tissue than in the synovium. Moreover, no differences were found, in the expression of the different wnt ligands, between the synovium isolated from control and β-cat^DEL^ mice under the steady state (Fig 9A). In opposite, in the cortical tissue the mRNA expression of wnt5a, wnt5b, wnt7a and wnt9a were reduced in β-cat^DEL^ compared to the control (Fig 9B).

In summary, we demonstrated that in the synovium only a few of the wnt ligand tested were present and the expression levels of those did not differ between control and β-cat^DEL^ mice. In contrast, most of the wnt ligands studied were present in the cortex and the expression of wnt5a, wnt5b, wnt7a and wnt9a was reduced in the β-cat^DEL^ cortices. These data reinforce the hypothesis that difference in phenotype observed in the different disease models can indeed, at least in part, be explained by differences in the expression of wnt ligands in the target tissues.

## Conclusions

In this study we demonstrated that: a) deletion of *β-catenin* in BMDCs did not influence their activation, maturation profile or cytokine expression under steady state conditions and upon *in vitro* activation with a TLR2 (MTB), TLR4 ligand (LPS) or collagen (CII); b) in the steady state, *β-catenin* deficiency in DCs did not affect the T cell homeostasis *in vivo*; c) loss of *β-catenin* in DCs did not alter the onset, progression or severity of CIA; d) deletion of *β-catenin* in CD11c^+^ cells resulted in a reduction of 20% in the percentage of splenic Tregs during CIA; e) synovial expression of the wnt ligands: wnt2b, wnt5a, wnt5b and wnt9a.

Based on the published literature, we initially hypothesized that *β-catenin* plays a critical role in the switch between the tolerogenic and immunogenic function of DCs and that the conditional deletion of *β-catenin* might lead to exacerbated CIA and a pro-inflammatory cytokine profile, with increased Th1 and Th17 cells and decreased Tregs. It has been shown that DCs play a role in the initiation of arthritis (6) and might have therapeutic potential to treat RA, since administration of semi-mature or tolerogenic DCs can inhibit CIA (7–9). In this study, we observed a small decrease in Treg frequency in CIA β-cat^DEL^ mice, but we did not find differences in the percentages of Th1 and Th17, nor in the pro-inflammatory cytokines expressed by these cells. These data suggest that the *β-catenin* pathway in DCs is not a major switch in the control of CIA. In contrast to CIA, in dextran sodium sulphate-induced IBD and EAE the deletion of *β-catenin* in DCs led to an exacerbation of disease severity and an increase in local inflammation [13–15]. On the other hand, activation of *β-catenin* signalling by stabilization of the protein using a β-catenin agonist or by TLR2 stimulation seems to protect mice from EAE [14, 15]. We hypothesize that the dissimilar consequences of deletion of *β-catenin* in CD11c^+^ for the development of IBD, EAE and CIA might at least in part be explained by the local tissue expression of wnt proteins and the DC-intrinsic gene machinery that lead to differential activation of wnt target genes in DCs present in each target tissue. In this study, we demonstrated that wnt2b, wnt5a, wnt5b and wnt9a are expressed in the synovial tissue and no differences were found between control and β-cat^DEL^ mice. However, in cortical tissue beside those ligands also wnt3, wnt7a, wnt8b are present. Interestingly, in the cortex expression of wnt5a, wnt5b, wnt7a and wnt9a was reduced in the β-cat^DEL^ cortices. These data suggest that the differences in phenotype observed in the different disease models can, at least in part, be explained by differences in the expression wnt ligands in the target tissues.

While further studies are needed to clarify this hypothesis, our findings highlight that *β-catenin* signaling in DCs may not be the general ‘master switch’ of a tolerogenic DC phenotype as it appears not capable to influence the course and severity of autoimmune arthritis.

## Supporting Information

S1 Fig Gating strategy to identify Th cell subsets based on their chemokine receptor profile. Lymphocytes were gated according to their forward light scatter (FSC) and side light scatter (SSC). Cell debris and doublets were excluded, and the cells were divided based on their CD3 (BV785) expression. NK T cells were identified within the CD3^-^ population based on their NK1.1 (A700) expression. CD3^+^ cells were further divided into CD8^+^ (PE-Cy5) and TCRδγ^+^ (BV605) cells and the double-negative population, containing mostly CD4^+^ T cells, was further resolved based on CD4 (V500) and CD25 (PerCP-Cy5.5) expression. CD25^high^CD4^+^ cells (Tregs) were excluded. The remaining population was gated (1) on CXCR5 (FITC) and PD-1 (BV421) expression to identify Tfh or gated (2) on CD4 and CD62L (APC-Cy7). Naïve CD62L^+^ T cells were excluded, and effector T cells (CD4^+^CD62L^-^) were further divided based on their CCR6 (PE) expression. CCR6^+^ and CCR6^-^ cells were further gated based on their expression of CCR4 (APC) and CXCR3 (Pe-Cy7). From these, on the CCR6^+^ compartment we could identify: Th17 (CCR6^+^ CCR4^+^CXCR3^-^) and Th17.1 (CCR6^+^CCR4^-^CXCR3^+^) and on the CCR6^-^ compartment the Th2 CCR6^-^CCR4^+^CXCR3^-^ and Th1 CCR4^-^CXCR3^+^.
(TIF)
