# Supplementary figures and images for: Dendritic Cell-Specific Deletion of β-Catenin Results in Fewer Regulatory T-Cells without Exacerbating Autoimmune Collagen-Induced Arthritis
Source: PLoS One. 2015 Nov 20;10(11):e0142972. doi: 10.1371/journal.pone.0142972 (PMC4654567; doi:10.1371/journal.pone.0142972)

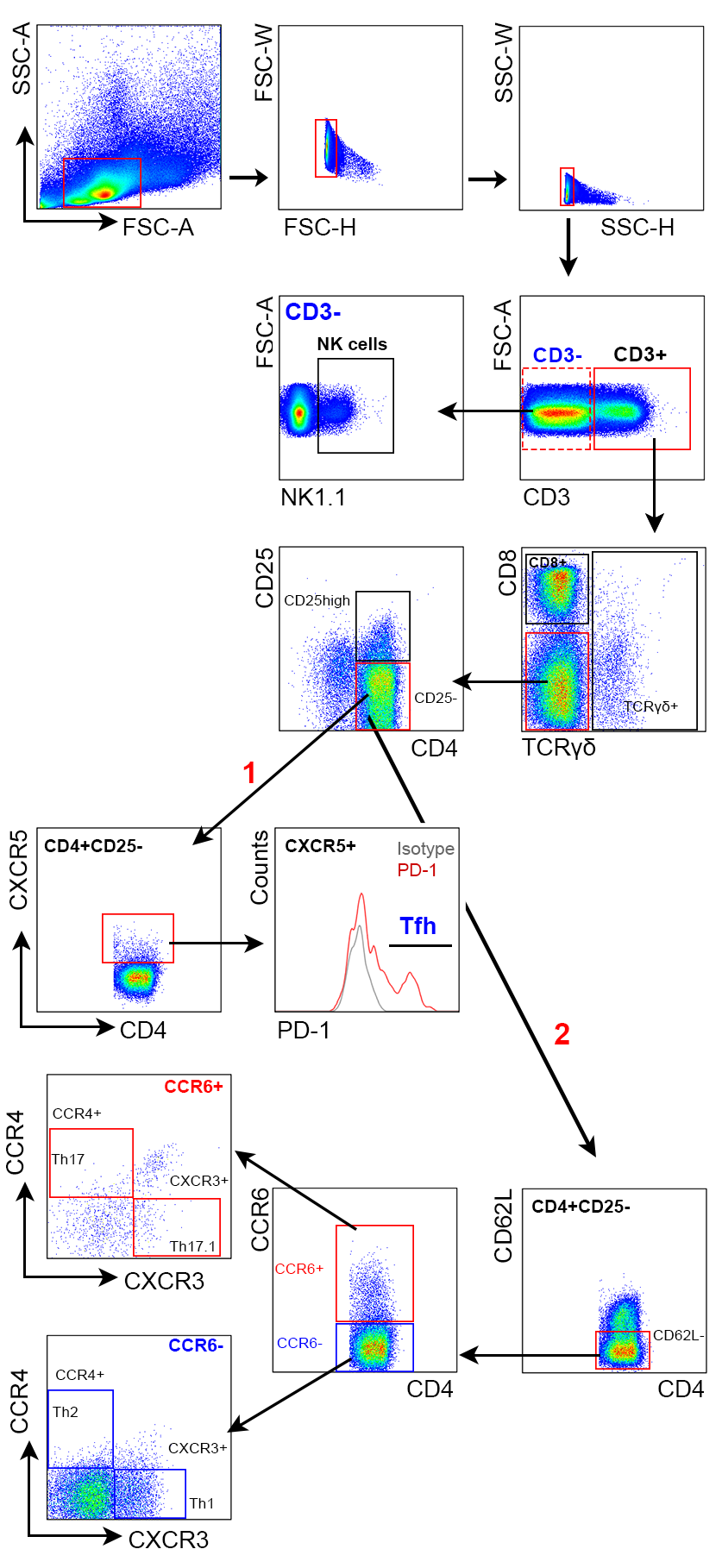

Supplement: S1 Fig — Lymphocytes were gated according to their forward light scatter (FSC) and side light scatter (SSC). Cell debris and doublets were excluded, and the cells were divided based on their CD3 (BV785) expression. NK T cells were identified within the CD3- population based on their NK1.1 (A700) expression. CD3+ cells were further divided into CD8+ (PE-Cy5) and TCRδγ+ (BV605) cells and the double-negative population, containing mostly CD4+ T cells, was further resolved based on CD4 (V500) and CD25 (PerCP-Cy5.5) expression. CD25highCD4+ cells (Tregs) were excluded. The remaining population was gated (1) on CXCR5 (FITC) and PD-1 (BV421) expression to identify Tfh or gated (2) on CD4 and CD62L (APC-Cy7). Naïve CD62L+ T cells were excluded, and effector T cells (CD4+CD62L-) were further divided based on their CCR6 (PE) expression. CCR6+ and CCR6- cells were further gated based on their expression of CCR4 (APC) and CXCR3 (Pe-Cy7). From these, on the CCR6+ compartment we could identify: Th17 (CCR6+ CCR4+CXCR3-) and Th17.1 (CCR6+CCR4-CXCR3+) and on the CCR6- compartment the Th2 CCR6-CCR4+CXCR3- and Th1 CCR4-CXCR3+. (TIF) [file pone.0142972.s001.tif]
